# Bilateral Deficit and Bilateral Performance: Relationship with Sprinting and Change of Direction in Elite Youth Soccer Players

**DOI:** 10.3390/sports8060082

**Published:** 2020-06-03

**Authors:** Giampiero Ascenzi, Bruno Ruscello, Cristoforo Filetti, Daniele Bonanno, Valter Di Salvo, F. Javier Nuñez, Alberto Mendez-Villanueva, Luis Suarez-Arrones

**Affiliations:** 1 Faculty of Sport, University of Pablo de Olavide (UPO), 41013 Sevilla, Spain; 2 Department Football Performance & Science, Aspire Academy, Doha 22287, Qatar; daniele.bonanno@aspire.qa (D.B.); valter.disalvo@aspire.qa (V.D.S.); 3 Italy School of Sport Sciences and Exercise, Faculty of Medicine and Surgery, “Tor Vergata” University, 00133 Rome, Italy; bruno.ruscello@alice.it (B.R.); cris.86@hotmail.it (C.F.); 4 School of Sports and Exercise Sciences, “San Raffaele” University, 00166 Rome, Italy; 5 Department of Industrial Engineering, Faculty of Engineering, “Tor Vergata” University, 00133 Rome, Italy; 6 LUISS Sport Lab, LUISS University, 00197 Rome, Italy; 7 Physical Performance and Sport Research, Pablo de Olavide University, 41013 Sevilla, Spain; fjnunsan@upo.es (F.J.N.); ljsuamor@upo.es (L.S.-A.); 8 Qatar Football Association, Doha 22287, Qatar; amendezvillanueva@yahoo.com; 9 Performance Department, FC Basel 1893, 4052 Basel, Switzerland

**Keywords:** performance analysis, BLD, bilateral testing, unilateral testing, power

## Abstract

The purpose of the study was to examine the differences in bilateral deficit (BLD) at different loadings during the half-squat jump (SJ) and horizontal countermovement jump (HCMJ) to determine if there is a relationship with linear sprint or change of direction (COD). The second goal was to check if fast players were more powerful in SJ and HCMJ than slow players in bilateral performance (BP). Twenty-seven male youth soccer players participated in the study. Players were divided in two groups, faster and slower, according to their sprint performance (10 and 40 m). BLD average power with body weight (BW) and 25%BW were significantly higher than 50%BW (*p* < 0.01). BLD during HCMJ was significantly higher than BLD during SJ with BW, 25%BW and 50%BW (*p* < 0.01). There were no statistical relationships between BLD and sprint or COD performance (*p* > 0.05). Fast players showed significantly higher SJ power with all the different loads and HCMJ than slow players (*p* < 0.01), and fast players lost more time executing COD-90° than slow players (*p* < 0.01). There were no statistical differences between fast and slow players in BLD. BLD seems to be dependent on motor task, contraction type and load and could not be a proper measure to estimate sprint and COD performance. Faster players are confirmed to be more powerful players than slow players, and decrements in COD could be a key benchmark to identify deficit between linear and COD performance.

## 1. Introduction

Strength motor task assessments can be performed with bilateral (BL) or unilateral (UL) exercises. When the sum of the force or power produced by each limb acting in the UL condition is larger than the force or power generated in the BL condition, it is defined as the bilateral deficit (BLD) phenomenon. [1,2,3]. The occurrence of the BLD has been showed in different contraction types: isokinetic [4,5], isometric [6] and specific sport-related motor tasks [7]. Nevertheless, the elements that influence the BLD are not well defined in the literature. Several researchers have focused on the potential mechanisms explaining this phenomenon, such as neural aspects [8], changes in motor unit recruitment [9], force–velocity relationship [10], limb dominance [8], training preference [8] and the postural behaviors in the UL jump [11]. Consequently, it seems plausible to consider the BLD as a multifactorial phenomenon rather than due to a single factor.

Due to the diversity of the contractile elements and mechanisms that seem to be involved, the BLD emerges significantly in all human movements, particularly when multiple joints are engaged [1]. On the other hand, when the action is accomplished by a single joint, the relevance of the BLD is less evident [12]. Conversely, when the sum of the force or power produced by each limb acting in the UL condition is smaller than the force or power generated in the BL condition, it is defined as the bilateral facilitation (BLF) [8,13].

Considering all of the above, the selection of assessment procedures suitable to describe the player strength profile plays a crucial role in defining the BLD. A previous study by Bishop et al. [14] reported that a larger BLD in countermovement jump (CMJ) was associated with a faster change-of-direction speed (CODS) during the 505 (r = −0.48 and r = −0.53, for left and right legs respectively) but not with linear speed. Furthermore, Bračič et al. [15] showed a significant correlation between a smaller BLD, high peak force production and a higher total impulse (r = −0.63; *p* < 0.01), suggesting that the 60 and 10 m sprint performances were associated with a lower BLD. Consequently, we can assume that lower values of BLD are associated more with the performance of tasks where BL actions are the primary requirement (e.g., volleyball, rowing, weightlifting, etc.); conversely, a higher value of BLD can have more potential benefits during the performance of tasks that require UL actions [1] (e.g., soccer, football, basketball and hockey, among others). Nevertheless, numerous researchers are trying to explain BLD in different sports [10,14,15], but to date there is a lack of studies regarding BLD applied in soccer players. 

Soccer is a physically demanding sport where endurance, strength, explosive power and repeated sprint abilities have been shown to be important factors in determining success [16,17,18,19,20,21,22,23]. During soccer training and matches, lower-limb strength and power are crucial for executing different specific actions such as accelerating, decelerating, changing direction or sprinting. Straight sprinting is the most frequent action in goal situations in soccer [24]. Increasing the concentric explosive strength and reactive strength improves the stride length and ground contact during the first meters in a sprint [25]. Horizontal and vertical power are important factors to explain sprint performance in soccer players. Previous studies have been demonstrating strong correlations between squat, sprinting and jumping performances in elite soccer players [26,27]. In addition, horizontal hop distance is a predictor of lower body strength and power in soccer athletes [28]. Squat and vertical jump movements have been systematically employed to explain sprint performance, showing substantial associations with performance in short sprints [26]. In contrast, previous investigations have not found significant relationships between strength, jump and sprint times [29,30]. Straight sprinting is considered one of the key potentials for the effectiveness of COD performance [31], and a recent study showed statistical correlations between linear straight sprinting, COD 90° and 180° performance tests [32]. COD can be considered multifactorial, defined as the ability to decelerate, reverse or change movement direction and accelerate again [33,34]. The vast majority of actions or movements in soccer require 3-dimensional deceleration and acceleration, calling for rapid and agile CODs [35,36]. COD ability has been considered one of the most useful soccer-specific tools in training sessions [37]. Previous studies have shown improvements in COD due to eccentric overload training using flywheel and vibration exercises [35], back squat strength training [38] and UL and BL squat training with flywheel resistance exercises [39].

The relationship between UL strength training and COD in soccer has been largely investigated [40,41,42,43]. Recently, Núnez et al. [39] demonstrated that UL strength training seems to be more effective in improving COD abilities performance than the BL modalities. In addition, Gonzalo-Skok et al. [43] showed that one training session per week for eleven weeks of UL eccentric overload, in addition to vibration training, improved the linear speed, jump performance and COD ability more than the conventional strength training in youth soccer players. Furthermore, Gonzalo-Skok et al. [44] showed that with two training sessions per week, BLD increased more than in BL strength training in elite youth basketball players. Despite the existence of a large number of studies showing relationships between all these variables, straight linear sprinting, jumping performance, CODs and half-squat power are, for the most part, separate motor qualities; therefore, it is suggested that all of them should be tested and trained individually [32]. Despite the relevance of the connections among the BLD, COD abilities and soccer, there is a lack of investigations that have recently studied the phenomenon of BLD applied to young soccer players. Consequently, the aim of this study was to examine the differences between BLD at different loadings during half squat jumps (body weight (BW), 25%BW and 50%BW) and horizontal CMJ (HCMJ) and to determine if there was a relationship with linear sprint (10, 20, 30 and 40 m) and COD. The second goal was to examine if fast players were more powerful in squats (at different loadings) and horizontal jumps than slow players in bilateral performance (BP).

## 2. Materials and Methods

### 2.1. Experimental Design

An observational study was designed to verify the given hypotheses. The measures obtained by testing were considered to be dependent variables, whereas different factors, such as fast and slow players and different loadings, were set as independent ones.

### 2.2. Participants 

Twenty-seven young male soccer players from Aspire Academy Club’s U-17’s and 18′s Qatar National Team (Doha, Qatar) participated in this study (*n* = 27; age: 18.5 ± 0.6 years; height: 175.4 ± 15 cm; body weight 67.5 ± 14.8 kg). Within the sample there were 23 players that participated in the previous FIFA U-20 World Cup (Poland 2019). All subjects in the study had a minimum of 3 years’ experience in a professional football academy. Over the previous year, the players usually trained 5 to 7 times per week (six to eight training sessions, two strength-training sessions, one or two conditioning sessions, and two international club games every three weeks). Twenty-four players competed at the international level and may be considered elite level for their age group. Before undergoing assessment, all players with previous knee injuries or chronic lower limb injures did not fulfil the eligibility criteria and were not considered. The methodology used was approved by the Ethics Committee (Qatar Antidoping Lab, E2013000004) and conformed to the policy statement with respect to the Declaration of Helsinki. All participants and their parents were informed of the risk and benefits of the procedure and signed an informed consent for participation in the study.

### 2.3. Procedures

Data were collected during the preparation period for the FIFA-U20 World Cup. The experimental stage consisted of two different phases. On day 1, the participants underwent the strength assessments (squat jump at different loadings and horizontal jump). On day 2, the participants underwent all speed assessments: COD and linear sprinting. A recovery time of 72 h was granted between day 1 and day 2 in order to reduce the effect of fatigue. To avoid any circadian influences on testing, all the assessments were carried out at the same time each day. To ensure familiarization with the procedures, participants conducted trial tests over 10 sessions, spanning three weeks prior to testing. Strength testing was performed in an indoor gym (Aspire Academy, Doha, Qatar) where the environmental conditions were considered optimal in terms of stability and reproducibility (10.30 a.m., 21 ± 0.5 °C average temperature and 50% ± 2% relative humidity). COD and sprinting testing were performed on an artificial turf homologated for international competitions. Players wore soccer shoes usually adopted during competitions. Participants refrained from any heavy training in the two days prior to testing.

On testing day 1, BW was measured in order to compute BW25% and BW50%. During day 1, all players performed a general warm up comprising 5 min cycling, 3 min ballistic stretching, 1 × 8 repetitions of back squats (Smith Machine) in bilateral and UL execution and 1 × 3 repetitions HCMJ at 50%, 70% and 90%, approximate capacity in BL and UL execution. During day 2, all players completed a general warm up comprising 5 min bicycle, 3 min ballistic stretching, run for 30–40 m at gradually increasing speeds and acceleration over 10–20 m, including taking off at maximum effort and sharp COD at 90°.

### 2.4. Squat Jump Test (SJ)

All the strength assessments during squatting were performed in three different approaches: (a) BL execution, (b) UL execution pushing with left leg (UL_L_) and (c) UL execution pushing with right leg (UL_R_). All assessments were recorded using a linear encoder (SmartCoach^TM^, EuropeAB, Stockholm, Sweden). After the general warm up, each player performed three repetitions of BL, UL_L_ and UL_R_ SJ with the Smith Machine (Multipower, Technogym tm, Gambettola, Italy) as the specific warm up. For testing, players performed three repetitions of BL followed by three UL_L_ and UL_R_ SJ. During the BL, participants were positioned at 90° knee angle [45] using a professional goniometer (Baseline^®^ Measurement, White Plains, NY, USA) and then performed the SJ a maximal ballistic push off (positive phase, upward extension). During the UL_L_ and UL_R_, participants performed one-leg squats at 90° knee angle [46,47], and each player carried out a maximal ballistic push off [48]. Thirty seconds of recovery was set between BL, UL_L_ and UL_R_ repetitions, and two minutes of recovery was set between the three different loadings. The testing sequence involved lifting the bar (7.3 kg) of the Smith Machine without weights for the BW trial, and additional loading for the BW25% and the BW50% trials. The best mean power (avg. power) and the best peak power trials with each load were considered for the consequent statistical analysis [49,50].

### 2.5. Horizontal Countermovement Jump (HCMJ)

Participants stood on the starting point as per standing long jump. Hands were fixed on the hips, and it was requested to hop and land, remaining stationary when landing (no additional hops were allowed) until the test leader recorded the result [51]. If the players failed the landing phase the test was considered invalid and was repeated. Subjects performed three jumps. Recovery time between jumps was 1 min. The best performance in terms of horizontal distance (cm) was used for the subsequent analysis.

### 2.6. Horizontal Countermovement Jump Single Leg (HCMJ_L_ and HCMJ_R_)

Participants began standing, balancing on a single leg (the one to be tested) and then requested to hop as far as possible landing on the same leg. Hands were fixed on the hips, and opposite leg swing was permitted. As per HCMJ, subjects were requested to hop and land, remaining stationary when landing (no additional hops were allowed) until the result was noted [51,52]. If subjects failed the landing phase the test was considered invalid and was repeated. Subjects performed a total of three jumps with each leg. Recovery time between jumps was 1 min. The best performance with each leg in terms of horizontal distance (cm) was used for the subsequent analysis. 

### 2.7. Bilateral Deficit (BLD)

The BLD was computed according to the following formula (Howards et al. [8]):

(1) $$\mathrm{BLD}\;(\%)=[100\;\times \;(\frac{\mathrm{bilateral}}{\mathrm{Right}\;\mathrm{unilateral}\;+\;\mathrm{Left}\;\mathrm{unilateral}\;})]\;-100,$$

This determines the ratio between the BL execution and the sum of the UL ones during ballistic strength tasks (e.g., SJ and HCMJ). 

### 2.8. Sprint Assessment (10, 20, 30 and 40 m)

Participants performed a maximal 40 m sprinting test. Two trials were considered. Split 10, 20 and 30 m times were also recorded (Smart-Fusion Sport^TM^, Brisbane, Australia). Players started each trial from a standing still position with their front foot 0.5 m behind the first timing gate. They self-administered their starting. They were encouraged to perform the sprint as fast as possible until the last gate [52]. Each trial was separated by two minutes of passive recovery, and the best performance was used for the subsequent analysis.

### 2.9. Change of Direction Assessment (COD-90°)

Participants performed a maximal 10 + 10 m “L” sprinting test (with a 90° change of direction) with left and right turns. Players started each trial from a stationary standing position with their front foot 0.5 m behind the first timing gate (Smart-Fusion Sport^TM^, Brisbane, Australia). They self-administered their starting. They were encouraged to perform the sprint as fast as possible until the last gate [53]. Two trials for each side were considered. Each trial was separated by two minutes of passive recovery, and the best performance was used for the subsequent analysis [54]. The best result for the right and for the left COD-90° test was compared to the fastest 20 m straight-line sprint time. The loss of speed caused by executing COD-90° (DEC-COD) was calculated as a percentage using a formula as in previous studies [32]:

[(T COD-90° × 100)/T 20 m] − 100(2)

T COD-90° = Time in COD-90° test
T 20 m = Time in maximal 20 m sprinting test

### 2.10. Data Analysis 

Data are presented as the mean and standard deviation (M ± SD) and the range. The assumption of normality was assessed using the Kolmogorov–Smirnov test. The intraclass correlation coefficients (ICCs) for measures were provided as indices of relative reliability of the measurements. A correlation matrix (r) describing the association of several variables was provided. To find significant differences among the collected measures performing different testing (dependent variables), the different loadings (BW, 25%BW, 50%BW) and HCMJ were set as independent variables, and analysis of variance (ANOVA) was then performed. Subsequent post hoc tests performed with Bonferroni’s correction of significance level were provided. The value of statistical significance was accepted as *p* ≤ 0.05. IBM SPSS 25.0 for Windows was used to analyze and process the collected data. The corresponding *p* values were provided for each analysis (*p* ≤ 0.05). To assess how each sprinting performance parameter (10 and 40 m) could behave in relation to the strength measures, the sample was divided in two groups: “fast” and “slow” [31,32,33,34,35,36,37,38,39,40,41,42,43,44,45,46,47,48,49,50,51,52,53,54]. The two groups were created by computing the mean of the overall sample, for each sprinting distance, and then allocating the players to the respective groups (below and above the mean). To investigate the possible differences between the two groups “fast” and slow” in the sprinting performance, a t-test for independent samples was performed showing significant differences between groups (*p* ≤ 0.05). The effect size (ES) was determined, and the threshold values for Cohen’s ES statistics were classified as trivial (0.0–0.19), small (0.2–0.59), moderate (0.6–1.1), large (1.2–1.9) and very large (>2.0) [55].

## 3. Results

The ICCs as relative reliability of the measurements collected during testing were bilateral deficit avg power (0.76 (0.52–0.89), *p* < 0.01), bilateral deficit peak power (0.14 (−0.33–0.53)), sprint time (0.99 (0.98–0.99), *p* < 0.01) and change of direction time (0.88 (0.73–0.95), *p* < 0.01).

Descriptive values of parameters measured and computed are shown in Table 1 and Table 2. BL power and BL HCMJ were significantly higher than UL power and UL HCMJ (right or left leg interchangeably, *p* < 0.01).

**Figure 1:**
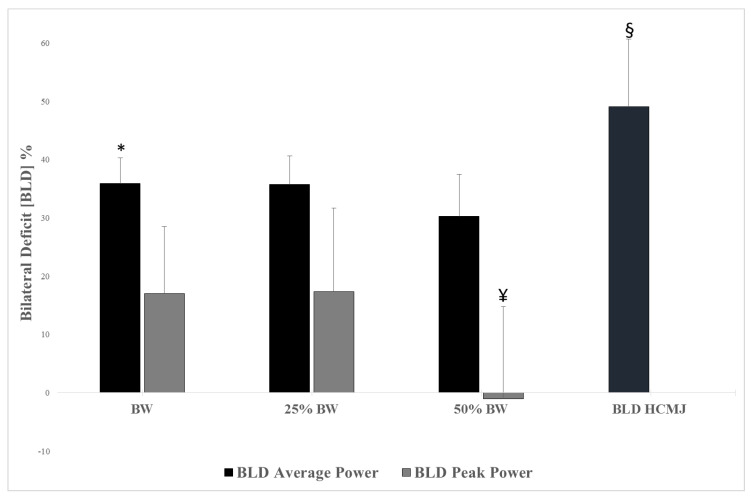
Bilateral deficit (BLD) behaviors at three different loadings during squat jump and horizontal countermovement jump. *: Significantly different vs. 50% body weight (BW) (*p* < 0.05); ¥: significantly different vs. BW and 25%BW (*p* < 0.01); §: significantly different vs. all the others (*p* < 0.01).

The BLDs during SJ with different loadings (BW, 25%BW and 50%BW) and during HCMJ are illustrated in Figure 1. BLD_AvgPower_ with BW and 25%BW were significantly higher than 50%BW (*p* < 0.01). BLD_PeakPower_ with BW and 25%BW were significantly higher than 50%BW (*p* < 0.01). BLD during HCMJ was significantly higher than BLD avg and peak power during SJ with BW, 25%BW and 50%BW (*p* < 0.01). There are no statistical relationships between BLD in SJ and sprint or COD performance (*p* > 0.05), except a moderate correlation between SJ BLD 25%BW_Avg_ and COD-DEC on the right side (r = −0.448). There are no statistical relationships between BLD in HCMJ and sprint or COD performance (*p* > 0.05). 

The differences between fast and slow players are shown in Table 3. Fast players showed significantly higher BP_AvgPower_ with all different loads compared to slow players (except with 25%BW in the fast players in 10 m) (*p* < 0.01, ES from 0.87 to 1.39). Fast players exhibited statistically higher HCMJ than slow players (*p* < 0.01, ES = −0.83; and ES = −0.73 for fast players in 10 and 40 m respectively). Fast players lost more time executing COD-90° than slow players (*p* < 0.01, ES = −1.30 and −1.16 for left and right leg, respectively, in fast players in 10 m; and *p* < 0.01, ES = −0.91 for left leg in fast players in 40 m). There were no significant differences between fast and slow players in COD-90° (*p* > 0.05). There are no statistical relationships between 10 m time and COD-90° (r = 0.46 (−0.01; 0.63), R^2^ = 0.22 (average time for left and right)), while a small association is detected between 40 m time and COD-90° (r = 0.58 (0.26; 0.77), R^2^ = 0.33 (average time for left and right)). There were no statistical differences between fast and slow players in BLD.

## 4. Discussion

The purpose of the study was to examine the differences in BLD at different loadings during half squat jump and horizontal CMJ to determine if there was a relationship with linear sprint (10, 20, 30 and 40 m) or COD, and to check if fast players were more powerful in squat jumps and horizontal jumps than slow players in BP. To our knowledge this is the first study that analyzed elite young soccer players’ BP and BLD in SJ and HCMJ in relation to the linear sprinting and COD ability. The main findings of this study are the following: (i) BLD with low loads (i.e., BW, 25%BW) was significantly higher than BLD with high loads (i.e., 50% BW); (ii) BLD during HCMJ was significantly higher than BLD during SJ at different loads; (iii) no relationships were found between BLD and SJ, HCMJ, sprint and COD performance; (iv) fast players showed significantly higher BP_AvgPower_ and HCMJ than slow players and (v) fast players lost more time executing COD-90° than slow players. 

Psycharakis et al. [56] studied BLD in SJ as well as CMJ with BW and 10%BW in recreationally active participants. They showed that BLD was present in SJ and CMJ at both loaded and unloaded conditions, but the additional load did not have a significant influence on the magnitude of BLD in both tests. In our case, present findings show a significantly higher percentage of BLD in SJ with BW and 25%BW than 50%BW (−16%). Based on these results, we speculate that, as we increase the load, BLD is reduced due to the lack of UL force in order to produce optimal levels of power. Moving this concept to a practical application, it seems that SJ with 50%BW could be considered a high demanding load in this ballistic strength task for this specific population of elite youth soccer players. Along this line, BLD computed in HCMJ was an order of magnitude greater (from 28% to 40%) than the ones developed in the selected vertical task with different loads. This finding suggests how BLD could be considered a sensible phenomenon across the different jump tasks [14]. Furthermore, in line with previous studies [1,4,5,6,7], our results demonstrate how BLD seems to be dependent on the motor task, contraction type and load.

The relationship between BLD jump and sprint performance has been previously examined in scientific literature. Bracic et al. [15] highlighted that the sprinters with higher CMJ BLD produced a lower total impulse of force on the block and lower block velocity, which were related with 60 and 100 m sprint performances. Showing the relationship between these two variables, the study suggested that elite sprinters with high BLD in CMJ should have a lower performance during sprinting. In contrast, the present findings shows no associations between the sprinting performance with SJ BLD at different loadings and HCMJ BLD in elite young soccer players. Along the same line, Bishop et al. [14] did not identify any relationships between BLD and sprint performance over both 10 and 30 m. Therefore, the findings in the scientific literature cannot conclude that changes in the BLD of athletes may have an influence on sprint performance.

In this study, no relationships between both BLD (SJ and HCMJ) and CODs variables have been observed, except a moderate correlation between SJ BLD 25%BW_AvgPower_ and COD-DEC in the right leg (r = −0.448). Our findings are different from those previously reported by Bishop et al. [14], which reported a moderate relationship between BLD in CMJ height and COD (expressed as deficit). A possible explanation for this discrepancy could be that the strength assessments used in our study differed from the ones by Bishop et al. [14] in the contraction type (concentric (SJ) vs. eccentric–concentric (CMJ)), and in the type of movement (horizontal (HCMJ) vs. vertical (CMJ)). Likewise, the COD tests used in the two studies were different (L-test 90° vs. 505), which required different cutting COD angles (90° vs. 180°) and starting type (standing vs. flying). Consequently, the differences found in the BLD between the present study and one by Bishop et al. [14] might be attributed to factors related to task, physiology and neuro physiology [12], which could provide different responses, whereas different testing and measurement procedures are applied.

Linking the previous findings to the COD performance, present results show no significant differences between the “faster” and “slower” players in COD-90° (*p* > 0.05). This result suggests that “faster” players over 10 and 40 m are not necessarily fast in COD-90°. Although both abilities have been considered as fundamental for football performance [24], our results with elite young soccer players do not demonstrate a clear relationship between linear sprint and COD performance (r from 0.46 to 0.58; R^2^ from 0.22 to 0.33). Therefore, based on the results of the present study, linear sprinting and COD are separate motor qualities and should be specifically assessed and trained. In addition to this, our results demonstrate how the “slower” players over 10 m showed a significantly lower COD-DEC in left- and right-side executions (ES = 1.30 and ES = 1.16) than “faster” players. Similarly, the “slower” players over 40 m showed a significantly lower DEC-COD in left side execution than the “faster” players (*p* < 0.04, ES = 0.91). Present results are in line with the recent study of Suarez-Arrones et al. [32] in which the fastest players lost more time performing the COD than their slower counterparts. As a practical application, in cases where athletes present a high DEC-COD, coaches should prescribe specific COD training in order to reduce the percentage of decrement. 

Horizontal and vertical power are important factors to explain sprint performance in soccer players [26,27]. While previous research showed statistical associations between squat strength/power, horizontal and vertical jump with sprint performance [26], other studies presented contradictory findings with no relationships between these parameters and sprint times [29,30]. To date, no study has investigated in elite young soccer players if fast players are more powerful in SJ and HCMJ than slow players. Our results show how “faster” players exhibited significantly higher BP_AvgPower_ and HCMJ than their “slower” counterparts, supporting the concept that improvements in lower body strength/power would benefit sprint performance in elite young soccer players. By contrast, there were no statistical differences between fast and slow players in BLD. Due to dissociations between BLD (SJ, HCMJ) and sprint performance and the absence of differences between fast and slow players in BLD, we can state that BLD is not a useful parameter to understand or estimate sprinting and COD performance in elite young soccer players.

### Limitations

This study has been carried out with the aim to analyze BLD and BP during different strength tasks and to analyze the relationships between sprinting and COD performance. For this reason, UL performance was not distinctly considered. Therefore, further research must take into consideration UL performance during strength tasks, and eventually the inter-limb asymmetries, in order to clarify the relationship between those parameters. Due to the importance that BLD could have in the identification of the neuromuscular profile in elite young soccer players, additional tests and kinetic parameters to those used in the present study should be examined.

## 5. Conclusions

Based on the present results, BLD seems to be dependent on the motor task, contraction type and load, and could not be a proper measure to predict or estimate sprint and COD performance in elite young soccer players. Faster players have confirmed to be more powerful players than slow players, and DEC-COD has proven to be a key benchmark for the purpose to identify deficits between linear and COD performance. 

## Figures and Tables

**Table 1:** Squat jump bilateral (B) and unilateral (L/R) tests performed with body weight (BW), 25% body weight (25%BW) and 50% body weight (50%BW).

Variables	Mean ± SD	Range	
		Max	Min
BL Average Power (W)			
BW	937.9 ± 114.2 **	1157.7	728.9
25%BW	992.6 ± 159.7 **	1529.6	798.1
50%BW	1064.4 ± 170.7 **	1593.1	838
UL Average power (W)			
BW	749.7 ± 92.3	978.1	587.1
25%BW	772.5 ± 107.3	1107.7	617.1
50%BW	755.9 ± 99.3	955.6	578.7
UR Average power (W)			
BW	715.4 ± 86.7	917.2	547.3
25%BW	765.1 ± 94.8	971.8	647.7
50%BW	768.5 ± 105.2	987.7	585.1
BL Peak power (W)			
BW	2921.9 ± 366.2 **	4382	1265
25%BW	2962.6 ± 533.6 **	4814	2310
50%BW	3042.1 ± 534.4 **	4686	2333
UL Peak power (W)			
BW	1845.9 ± 329.9	2551.4	1348.2
25%BW	1834.1 ± 352.1	2731	1399.7
50%BW	1730.1 ± 356.9	2384	1024.4
UR Peak power (W)			
BW	1712.5 ± 295.9	2370.4	1209
25%BW	1766.8 ± 289.3	2524.3	1163.9
50%BW	1761.9 ± 362.8	2652.2	883.7

BL = bilateral; UL = unilateral left; UR = unilateral right; W (watts); ** Significantly different vs. unilateral power. *p* < 0.01.

**Table 2:** Sprinting, change of direction (COD) and horizontal countermovement jump test (HCMJ).

Variables	Mean ± SD	Range	
		Max	Min
Sprinting (s)			
10 m	1.67 ± 0.06	1.84	1.53
20 m	2.91 ± 0.01	3.14	2.69
30 m	4.05 ± 0.12	4.37	3.77
40 m	5.19 ± 0.16	5.61	4.89
COD-90° (s)			
COD-Left	4.10 ± 0.11	4.30	3.89
COD-Right	4.14 ± 0.17	4.46	3.82
COD-DEC (%)			
Left	41.5 ± 3.8	49.1	54.3
Right	42.9 ± 5.4	33.4	35.5
HCMJ (cm)			
HCMJ-Bilateral	235.1 ± 12.9 **	271	208
HCMJ-Left	212.6 ± 7.8	232	190
HCMJ-Right	212.6 ± 11.2	239	189

COD-90° (s): change of direction time; COD-DEC (%): decrement (percentage) during change of direction; ** significantly different vs. unilateral countermovement jump (*p* < 0.01).

**Table 3:** Differences between fast and slow players in bilateral power, horizontal contra movement jump (HCMJ) and DEC-COD (%) (loss of speed caused by executing COD-90°).

Variables	Sprinting 10 m (s)			Sprinting 40 m (s)		
	Fast (*n* = 18)	Slow (*n* = 9)	ES	Fast (*n* = 17)	Slow (*n* = 10)	ES
BP_Avg Power_ (W)						
BW	966.1 ± 101.4	863.4 ± 102.8 *	−0.87 ± 0.69↓↓	980.9 ± 89.7	846.8 ± 95.3 **	−1.39 ± 0.70↓↓
25%BW	1010.8 ± 182.6	957.2 ± 99.8	−0.35 ± 0.62	1039.3 ± 176.3	909.4 ± 83.5 *	−0.90 ± 0.61↓↓
50%BW	1101.5 ± 179.5	958.4 ± 59.1 **	−1.03 ± 0.58↓↓	1116.2 ± 171.9	946.7 ± 74.2 **	−1.23 ± 0.60↓↓
BP_Peak Power_ (W)						
BW	2904.1 ± 598.2	2705.2 ± 284.8	−0.41 ± 0.61	2936.8 ± 607.1	2065.5 ± 260.4	−0.55 ± 0.60↓
25%BW	3052.5 ± 619.9	2846.3 ± 377.8	−0.39 ± 0.64	3159.1 ± 598.4	2667.8 ± 277.9 **	−1.01 ± 0.61↓↓
50%BW	3088.3 ± 750.8	2675.3 ± 220.3 *	−0.71 ± 0.57↓↓	3108.6 ± 764.1	2682.8 ± 238.4 *	−0.72 ± 0.58↓↓
Jump (cm)						
HCMJ	237.8 ± 12.9	227.5 ± 11.0**	−0.83 ± 0.70↓↓	238.1 ± 12.8	228.6 ± 12.5 **	−0.73 ± 0.71↓
COD 90°						
Left	4.08 ± 0.13	4.14 ± 0.09	0.50 ± 0.70	4.07 ± 0.12	4.17 ± 0.09	0.86 ± 0.73↓
Right	4.12 ± 0.18	4.17 ± 0.17	0.29 ± 0.72	4.10 ± 0.18	4.23 ± 0.12	0.77 ± 0.71↓
COD-DEC (%)						
Left	42.7 ± 3.4	38.2 ± 3.1 **	−1.30 ± 0.80↓↓	42.6 ± 3.5	39.1 ± 3.5 **	−0.91 ± 0.78↓↓
Right	44.3 ± 5.6	39.2 ± 2.5 **	−1.16 ± 0.66↓↓	43.7 ± 5.6	41.6 ± 4.9	−0.46 ± 0.75

BP: bilateral performance; AVG: average; (W): watts; BLD (%): percentage of bilateral deficit; BW: body weight; COD-DEC (%): decrement (percentage) during change of direction. ** Significantly lower vs. fast players. *p* < 0.01. * Significantly lower vs. fast players (*p* < 0.05). ↓↓ Significantly different. ↓ Substantial difference.
